# Associations Between Clinical Manifestations of SARS-CoV-2 Infection and HLA Alleles in a Caucasian Population: A Molecular HLA Typing Study

**DOI:** 10.3390/jcm13247695

**Published:** 2024-12-17

**Authors:** Bogusław Tymoniuk, Maciej Borowiec, Joanna Makowska, Emilia Holwek, Joanna Sarnik, Filip Styrzyński, Izabela Dróżdż, Andrzej Lewiński, Magdalena Stasiak

**Affiliations:** 1 Department of Immunology and Allergy, Medical University of Lodz, 251 Pomorska St., 92-213 Lodz, Poland; boguslaw.tymoniuk@umed.lodz.pl; 2 Department of Clinical Genetics, Medical University of Lodz, 251 Pomorska St., 92-213 Lodz, Poland; maciej.borowiec@umed.lodz.pl (M.B.); izabela.drozdz@umed.lodz.pl (I.D.); 3 Department of Rheumatology, Medical University of Lodz, 113 Zeromskiego Str., 90-549 Lodz, Poland; joanna.makowska@umed.lodz.pl (J.M.); joanna.sarnik@umed.lodz.pl (J.S.); filip.styrzynski@umed.lodz.pl (F.S.); 4 Central Clinical Hospital, Medical University of Lodz, 251 Pomorska St., 92-213 Lodz, Poland; e.holwek@csk.umed.pl; 5 Department of Endocrinology and Metabolic Diseases, Medical University of Lodz, 281/289 Rzgowska St., 93-338 Lodz, Poland; andrzej.lewinski@umed.lodz.pl; 6 Department of Endocrinology and Metabolic Diseases, Polish Mother’s Memorial Hospital-Research Institute, 281/289 Rzgowska St., 93-338 Lodz, Poland

**Keywords:** human leukocyte antigen, HLA, COVID-19, SARS-CoV-2, autoimmune, clinical course, risk, severe

## Abstract

**Background and Objectives**: Severe COVID-19 still constitutes an important health problem. Taking into account the crucial role of HLA in immune reactions, evaluation of the impact of HLA on COVID-19 risk and clinical course seemed necessary, as the already available data are inconsistent. The aim of the present study was to compare the HLA profiles of patients with symptomatic SARS-CoV-2 infection and a healthy control group, as well as to compare HLA allele frequencies in patients with severe and non-severe courses of COVID-19. **Materials and Methods**: HLA classes were genotyped using a next-generation sequencing method in 2322 persons, including 2217 healthy hematopoietic stem cell potential donors and 105 patients with symptomatic COVID-19. **Results**: Symptomatic course of SARS-CoV-2 infection appeared to be associated with the presence of HLA-A*30:01, B*44:02, B*52:01, C*05:01, C*17:01, and DRB1*11:02, while HLA-C*07:04 and DQB1*03:03 seem to play a protective role. Moreover, we demonstrated that the severe symptomatic course of COVID-19 can be associated with the presence of HLA-B*08:01, C*04:01, DRB1*03:01, and DQB1*03:01, while HLA-DRB1*08:01 appeared to be protective against severe COVID-19 disease. **Conclusions**: Identification of alleles that are potentially associated with symptomatic SARS-CoV-2 infection as well as the severe course of COVID-19 broadens the knowledge on the genetic background of COVID-19 course and can constitute an important step in the development of personalized medicine.

## 1. Introduction

Since the end of 2019, the SARS-CoV-2 pandemic has spread all over the world, and COVID-19 has become the most urgently and thoroughly analyzed new infectious disease. According to the Associated Press Agency, COVID-19 has killed at least 7 million people in the world; however, the actual number of fatal cases could be much higher. The World Health Organization (WHO) declared the end of the COVID-19 pandemic as a global health emergency; however, it is clear that the virus has remained present in the whole world and is changing into new variants that could severely affect people’s health and lives for many decades to come [1]. A global COVID-19-related problem is associated not only with mortality and acute complications of the disease but also with an enormous problem of high frequency and severity of long-term COVID-19 consequences (long COVID disease), which constitutes a huge burden for healthcare systems in all countries. 

Taking into account the significant differences in the clinical course in affected patients, many studies have focused on finding risk factors of symptomatic SARS-CoV-2 infection as well as of the severe course of COVID-19. The importance of many clinical factors, including older age, obesity, and cardiovascular disorders, was postulated [2,3,4,5]; however, severe COVID-19 was frequently observed in patients without such clinical risk factors. Therefore, the significance of genetic predisposition was suspected. Genes postulated to be associated with SARS-CoV-2 infection susceptibility and COVID-19 severity included *MUC1* [6,7], *SLC6A20* [6,8,9], *LZTFL1* [6,8,9,10,11,12,13], *FOXP4* [13], *ABO* [8,10,13,14], *SFTPD* [15], *IFNAR2* [13], *ELF5* [6,14,16], *OAS1* [11,13,15], *DPP9* [11,13,17], *TYK2* [6,7,11,13], *TLR7* [18,19,20], *ACE2* [13,17]; however, many of these results still require further confirmation. 

Taking into account the importance of the human leukocyte antigen gene (*HLA*) in immune responses, the role of HLA alleles was also proposed. Correlations between class I and class II HLA alleles and severe acute respiratory syndrome caused by SARS-CoV-2 were previously reported [21]. Associations between HLA and a risk of the severe course of COVID-19 were detected in previous studies; however, the results presented by different authors are not consistent. The severe course of COVID-19 was reported to be associated with HLA-A*11:01 and C*04:01 in European patients [22,23], while in an Asian population, severe COVID-19 was observed in carriers of several alleles reported by different authors. However, the results are highly inconsistent, with examples of alleles being reported as protective in some studies and as correlates of severe COVID-19 in others [21,24,25,26]. On the other hand, some studies have not shown any significant effect of *HLA* alleles on the risk of the disease or its asymptomatic course [27,28]. A summary of available results on *HLA* and COVID-19 correlations derived from studies that applied high-resolution methods is presented in Table 1.

Discrepancies between studies may result from differences in the definition of disease phenotypes, in study populations, and from a limited sample size. Despite inconsistent results, the role of HLA-related genetic susceptibility in the development of symptomatic or asymptomatic SARS-CoV-2 infection, as well as in the risk of severe course of COVID-19, seems to be significant, and this aspect requires further studies, especially in the Caucasian population, which has been less thoroughly analyzed compared with the Asian one. Therefore, the aim of the present study was to compare the HLA profiles of patients with symptomatic SARS-CoV-2 infection and a healthy control group, as well as to compare HLA allele frequencies in patients with severe and non-severe courses of COVID-19. Determination of alleles associated with increased risk of symptomatic infection and severe disease course will broaden our knowledge of mechanisms of immune responses in SARS-CoV-2 infection as well as will constitute an important step in the application of personalized medicine for patients with SARS-CoV-2 infection, especially in those with significant clinical risk factors of severe course and death.

## 2. Materials and Methods

### 2.1. Study Groups and Control Group

A total of 2322 people were included in the study, which comprised 2217 healthy hematopoietic stem cell potential donors and 105 patients with symptomatic SARS-CoV-2 infection. The large size of the control group was necessary to avoid bias related to potential infection that may occur in the future in the currently healthy members of this group and to avoid bias related to random changes (increases or decreases) in the frequencies of some alleles in a smaller group. Additionally, such a large control cohort allowed us to minimize the risk that an occurrence of a particular allele could not be compared to the study group. In our study, the control group included 248 alleles, with 45 in locus *A*, 79 in locus *B*, 46 in locus *C*, 49 in locus *DRB1*, and 29 in locus *DQB1*. In such a large group, we can consider that the confounding effects were eliminated. During the recruitment of the control group, the ethnicity of individuals was verified, and only Caucasians were included.

In the second phase of the study, on the basis of the severity of COVID-19, the patient group was divided into two groups: (1) 67 patients with severe COVID-19 (SC group) and (2) 38 patients with symptomatic but not severe COVID-19 (non-SC group). The course of COVID-19 was assessed based on clinical, radiological, and/or laboratory findings according to criteria defined by the American Thoracic Society (ATS) [43]. Severe disease was defined as the presence of one of the major criteria or more than 3 of the minor ones. The major criteria included septic shock requiring administration of vasopressors or respiratory failure requiring mechanical ventilation. The minor criteria included respiratory rate > 30 bpm, multi-lobe infiltration, confusion/disorientation, uremia, leucopenia, thrombocytopenia, hypothermia, and hypotension requiring intensive fluid resuscitation. The non-severe disease group included symptomatic patients not fulfilling the ATS severity criteria.

### 2.2. SARS-CoV-2 Infection Diagnosis Procedure

Our previous study demonstrated significant differences between the accuracies of different PCR-based SARS-CoV-2 RNA detection tests [44]. The highest sensitivities were observed with MediPAN and DiaPlexQ tests [44], and these tests were applied in the current study to confirm SARS-CoV-2 infection by detecting the genetic material of the virus in nasopharyngeal swabs.

### 2.3. HLA Typing Procedures

Blood samples were collected from each patient in 2.6 mL EDTA tubes, and 200 µL of DNA was extracted using column-based DNA isolation (Sherlock AX Kit for DNA purification, A&A Biotechnology, Gdansk, Poland). Each sample was first subjected to lysis using a solution that broke cells and released DNA. The lysate was then transferred to a silica-based column in the presence of high salt concentrations. Such conditions promoted the binding of DNA to the silica membrane of the column. Contaminants like proteins, lipids, and other cellular debris were removed by washing the column with a series of wash buffers. Finally, the bound DNA was eluted from the silica membrane using a low salt elution buffer, resulting in the presence of purified DNA in the eluate. HLA typing was performed using next-generation sequencing (NGS). To type five HLA loci, we used a commercial MIA FORA NGS FLEX 5 HT HLA kit (Immucor Transplant Diagnostics, Inc., 35 Technology Drive South, Warren, NJ, USA). The basic set contained each of the class I genes—LA-A, HLA-B, and HLA-C—as well as the class II genes, HLA-DRB1 and HLA-DQB1. The extracted DNA was fragmented, and adapters were added to each fragment to prepare a library suitable for sequencing. Specific primers were used to amplify the HLA regions of interest, ensuring that the subsequent sequencing focused on these critical areas. The prepared DNA library was sequenced using NGS technology, producing a vast amount of data corresponding to the genetic makeup of the HLA genes. Alleles were assigned using the Sirona Genomics NGS alignment software (Ver.4.5 Linux Server, Sirona Genomics Inc., Mountain View, CA, USA), which applied two complementary informatics strategies to make genotyping calls, aligning the sequences to reference HLA genes and identifying any variants. This step helped determine the specific HLA alleles present in the sample.

### 2.4. Statistical Analysis

For statistical calculations, the Statistica 13.3 EN 64 BIT software was used (StatSoft Polska Sp. z o.o., Kraków, Poland). Statistical calculations were performed for all alleles in individuals possessing a given allele. Statistical significance of differences between groups was assessed using the chi-square test for groups including ≥5 participants and the Fisher’s exact test for small groups (one or both groups < 5 participants), with *p*-values of <0.05 considered significant.

### 2.5. Ethics Procedures

After a detailed explanation of the purpose and character of the study procedures, all the patients signed an informed consent form for their participation. The study was approved by the Bioethics Committee of the Medical University of Lodz (approval code RNN/126/20/KE).

## 3. Results

The mean age of the patients at the time of diagnosis of COVID-19 was 60.2 ± 13.93 years. In the group of patients with symptomatic COVID-19, males and females were represented in similar proportions (*p* = 0.07). In the SC group, there were 29 male patients and 38 female patients, while in the non-SC group, there were nine male and 27 female patients. Statistically significant differences were found in the frequency of HLA alleles between patients with COVID-19 and the control group, as well as between SC and non-SC groups. For the control group, a deviation of genotypic frequencies from the Hardy-Weinberg Equilibrium at each HLA locus was analyzed. On the basis of GENEPOP vs. 4.7.5 Hardy-Weinberg test, *p* values for HLA-A, B, C, DRB1, and DQB1 were 0.4428, 0.9006, 0.9482, 0.5317, 0.3989, respectively.

### 3.1. Comparison of COVID-19 Patients and Control Group

**Figure 1:**
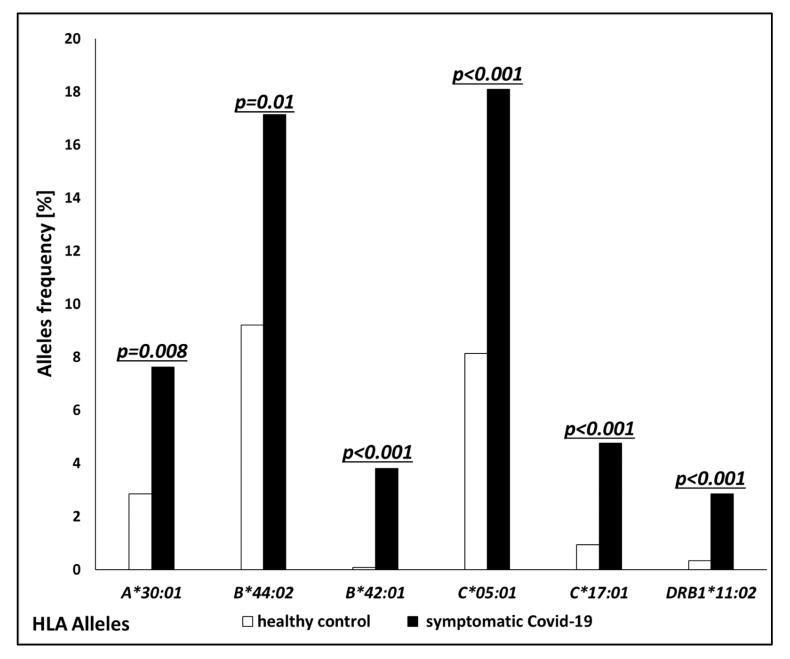
Frequencies (%) of human leukocyte antigen (*HLA*) alleles with statistically significant overrepresentation in COVID-19 patients (solid bars) compared to the control group (open bars).

Alleles with higher frequencies in COVID-19 patients compared to controls belonged to both MHC class I and class II. For MHC class I, the following alleles occurred more frequently in the COVID-19 group: HLA-A*30:01 (7.62% vs. 2.84%, *p* = 0.008), B*44:02 (17.14% vs. 9.22%, *p* = 0.01), B*42:01 (3.81% vs. 0.09%, *p* < 0.001), C*05:01 (18.1% vs. 8.14%, *p* < 0.001), and C*17:01 (4.76% vs. 0.95%, *p* < 0.001) (Figure 1). On the other hand, the frequency of HLA-C*07:04 was lower in the patients group compared to the controls (0.0% vs. 4.26%, *p* = 0.02). For MHC class II, the frequency of HLA-DRB1*11:02 was higher in COVID-19 patients compared to controls (2.86% vs. 0.34%, *p* < 0.001) (Figure 1), while the frequency of DQB1*03:03 was lower in the patients group (2.86% vs. 9.04%, *p* < 0.001). The odds ratios for all the analyzed alleles are presented in Table 2.

Two or more risk alleles were observed in 15 patients (39%) in the symptomatic COVID-19 group (thirteen patients with 2 alleles and two patients with 3 alleles). The most common combination was B*44:02-C*05:01, and in the two cases involving three alleles, the haplotype was A*30:01-B*44:02-C*05:01.

### 3.2. Comparison of Severe COVID-19 and Non-Severe COVID-19 Groups

**Figure 2:**
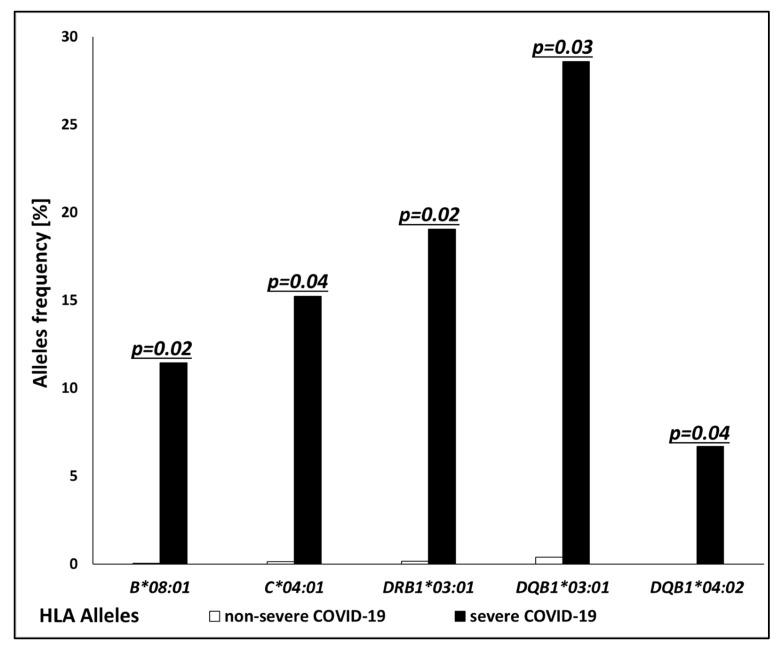
Frequencies (%) of human leukocyte antigen (HLA) alleles with statistically significant overrepresentation in severe COVID-19 patients (solid bars) compared to non-severe COVID-19 patients (open bars).

The mean ages of patients in the SC and non-SC groups were 62.8 ± 13.97 and 57 ± 13.45, with no significant differences between groups (*p* = 0.07). The alleles with higher frequencies in the SC group compared to the non-SC group belonged to both MHC class I and class II. For MHC class I, HLA-B*08:01 and C*04:01 occurred more frequently in the SC group than in the non-SC group (11.43% vs. 0.04%, *p* = 0.03 and 15.24% vs. 0.13%, *p* = 0.04), while for MHC class II, HLA-DRB1*03:01, DQB1*03:01, and DQB1*04:02 were more frequently present in the SC group (19.05% vs. 0.17%, *p* = 0.02; 28.57% vs. 0.39%, *p* = 0.03 and 6.67% vs. 0.0%, *p* = 0.04, respectively) (Figure 2). On the other hand, the frequency of HLA-DRB1*08:01 was significantly lower in the SC group compared to the non-SC group (0.22% vs. 0.95%, *p* = 0.01). The odds ratios for all the analyzed alleles are presented in Table 3.

Two or more risk alleles were observed in 19 patients (28%) with severe COVID-19 (eleven patients with 2 alleles, six patients with 3 alleles, and two patients with 4 alleles). The most common combination was C*04:01-DQB1*03:01. In cases involving three alleles, the haplotype was C*04:01-DRB1*03:01-DQB1*03:01, and for the two cases involving four alleles, both had the haplotype B*08:01-C*04:01-DRB1*03:01-DQB1*03:01.

## 4. Discussion

The COVID-19 pandemic has constituted one of the most important health problems all over the world in recent years. Knowledge of SARS-CoV-2 epidemiology, mechanisms of infection, immune reactions, as well as genetic and non-genetic risk factors has been increasing rapidly. Taking into account the crucial role of HLA in the majority of immune reactions, the evaluation of the impact of HLA on COVID-19 risk and clinical course seemed highly necessary, especially because the already available results for the Caucasian population are rather scant and inconsistent (Table 1). In our present study, we have demonstrated that the symptomatic course of SARS-CoV-2 infection is associated with the presence of HLA-A*30:01, B*44:02, B*42:01, C*05:01, C*17:01, and DRB1*11:02. Correlation of none of these alleles, except HLA-A*30:01, has been demonstrated before. The significance of HLA-A*30:01 was previously reported in a Vietnamese population [29], but this allele was considered protective against COVID-19. In our study of Caucasians, it was associated with a highly increased risk of symptomatic COVID-19 with an OR of 2.7.

HLA-A*30:02 and potentially A*30:04 were reported as being associated with SARS-CoV-2 infection in younger African Americans [34], while in our study in Caucasians, significance was found for HLA-A*30:01. In a study of British patients, HLA-DRB1*04:01 was associated with asymptomatic course [33], while in Americans HLA-B*15:01 seemed to play a protective role against symptomatic disease [32]. Our study did not confirm these findings, as we observed decreased frequencies of HLA-C*07:04 and DQB1*03:03 in COVID-19 patients.

Our study demonstrated a potentially increased risk of symptomatic COVID-19 in carriers of HLA-B*42:01. This finding can be supported by a demonstration of the same correlation between HLA-C*17:01 and symptomatic COVID-19, as there is a linkage disequilibrium between these alleles [45]. Therefore, in the cases in whom both alleles are present, their significance cannot be considered entirely independent. However, if either of them is present, it constitutes an independent risk factor. Our finding of a significance of HLA-B*44:02 and C*05:01 is also important, as these two alleles are also in linkage disequilibrium [45]; therefore, the demonstration of associations for both of them provides additional proof of their significance. The co-presence of these two alleles constituted the most common haplotype in our symptomatic COVID-19 group. A similar observation of the significance of HLA-B*44 was reported in a large observational study by Correale et al. [46]. On the basis of epidemiological analysis, the authors demonstrated that the prevalence of HLA-B*44 and C*01 correlates with COVID-19 spreading across Italy [46]. The consistency of our results and the results by Correale et al. strongly suggest a permissive role of HLA-B*44 towards SARS-CoV-2 infection.

Most previous studies analyzed potential correlations between HLA and the severe course of COVID-19 but not a risk of symptomatic infection. Therefore, our results on the potential risk of symptomatic SARS-CoV-2 infection are one of a few. Additionally, each of the available studies analyzed a different population. These facts resulted in high discrepancies between the reports. However, in regard to HLA and the risk of severe COVID-19 course, our observations of the significance of HLA-C*04:01 are consistent with several other studies, including those conducted in Spanish [22], Armenian [25], Indian [39], and German [23] populations. It should be underlined that HLA-C*04:01 was correlated with an increased risk of severe COVID-19 in both Asians and Caucasians. It was demonstrated that carriers of this allele were twice as likely to require mechanical ventilation compared to non-carriers [23]. Interestingly, a strong association between HLA-C*04:01 and subacute thyroiditis (SAT) has been demonstrated in Caucasians [47], in addition to the previously known correlation between SAT and HLA-B*35 [48]. Such a correlation can be suspected as there is a strong linkage disequilibrium between these two alleles [45]; however, we did not find an association between HLA-B*35 and COVID-19 in the present study. Other authors postulated this relationship in Whites, but the correlation did not reach statistical significance [34]. Nevertheless, due to strong linkage disequilibrium, both HLA-B*35:01 and C*04:01 may be considered to be associated with the severe course of COVID-19, and some inflammatory mechanisms can be common in COVID-19 and SAT. This speculation can be supported by the fact that SAT induced by SARS-CoV-2 infection as well as by COVID-19 vaccination was postulated to be HLA-dependent [48,49,50,51].

Our study demonstrated a potentially increased risk of severe COVID-19 in carriers of the HLA-DQB1*04:02 allele being exclusively present in patients with the severe course of the disease. This observation is consistent with the data reported by Lorente et al. [28], who found that DQB1*04 was associated with high mortality in Spanish patients with COVID-19.

In the present study, we demonstrated the importance of HLA-DQB1*03:01 in the severe COVID-19 course. This allele was reported to be linked to an increased risk of persistence of HBV, which is also an RNA virus [52]. Interestingly, the HLA-DQB1*03:01 allele is in linkage disequilibrium [45] with DRB1*11:02, which was found to be associated with symptomatic COVID-19 in the current study. Further studies on entirely independent large samples are necessary to find out which alleles and haplotypes are associated with symptomatic COVID-19 and which are associated with severe COVID-19, as in some of the studies, these groups may have overlapped as all severe COVID-19 patients could be considered either severe or symptomatic, depending on the study design. Except for HLA-C*04:01, none of the alleles found to be associated with the severe course of COVID-19 in Asians [21,24,26,29,30,31,37,38,39,41] were confirmed in our study.

The asymptomatic or mild course of the infection should also be thoroughly analyzed, as HLA alleles present in these patients may be associated with an ability to eliminate or inactivate the virus early. In our study, HLA-DRB1*08:01 was more frequent in patients with a mild course compared to the severe one. This allele was also reported to be associated with SARS-CoV-2 infection in the study by Amoroso et al. [53]. We also observed the presence of this allele in the symptomatic COVID-19 patients, but this association did not reach statistical significance, probably due to different group sizes, as our study included 105 symptomatic COVID-19 patients and 2217 healthy controls, while the study by Amoroso et al. included 219 COVID-19-positive patients and 40,685 healthy controls. Taking into account the fact that the proportions of the COVID-19-positive vs. control group sizes were 1:21 in our study and 1:185 in the study by Amoroso et al., the discrepancies between the results could be expected. Amoroso et al. also reported an increased frequency of DRB1*08 in fatal cases [53]. Our study did not analyze mortality, so we cannot compare the results. In another Italian study [36], HLA-DRB1*08 was exclusively present in four hospitalized patients. Discrepancies between our results and the results obtained by Littera et al. could be attributed to small sample sizes as well as to several other factors. Firstly, allele frequencies vary significantly in different parts of Italy [46], and HLA allele distribution in Sardinians may significantly further differ even from that of continental Italy, and the difference between Sardinians and Polish people may be much more relevant. Additionally, the differences in group selection should be taken into account, as Littera et al. compared asymptomatic COVID-19 patients to patients with mild or severe disease [36], while our study compared patients with symptomatic moderate disease to patients with severe, life-threatening disease. Our study did not include patients with asymptomatic COVID-19 patients.

Significant associations have been demonstrated between HLA-DRB1*03 and many autoimmune diseases (AIDs), including Graves’ disease [54,55] and Graves’ orbitopathy [56], Hashimoto’s thyroiditis [57], Addison’s disease [58], myasthenia gravis [59], Guillain–Barré syndrome [60], and systemic lupus erythematosus (SLE) [61]. Interestingly, in our present study, the HLA-DRB1*03:01 allele was found to be associated with the severe course of COVID-19, and this allele was four times more common in patients with severe COVID-19 than in those with a mild course of infection. Other researchers have found a link between SARS-CoV-2 infection and Guillain–Barré syndrome as an important post-infectious complication [62], and—as stated above—several studies have discovered a predisposition to Guillain–Barré syndrome in patients with the DRB1*03:01 allele [60]. HLA-DRB1*03 is a component allele of the AH8.1 haplotype (*HLA A1-B8-DR3-DQ2*) in Northern and Western Europeans. The genes between HLA-B*08 and DRB1*03 in this haplotype are often associated with autoimmune diseases [63]. We found that the presence of HLA-B*08:01 increased the risk of severe COVID-19 by more than eight times and, additionally, the HLA-B*08:01-C*04:01-DRB1*03:01-DQB1*03:01 haplotype was observed in both of our patients with co-presence of four risk alleles. Interestingly, we have recently demonstrated that in patients with GD, HLA-B*08:01 occurred most frequently together with DRB1*03:01 and DQB1*02:01 [54]. The frequent co-occurrence of these three alleles in autoimmune diseases was also observed by other authors [64,65,66,67,68]. The present finding of the significance of HLA-B*08:01 and DRB1*03:01 allows us to presume that one of the mechanisms of severe COVID-19 may be associated with autoimmune reaction. Several mechanisms that can explain the association of *HLA-DR/DQ* alleles with autoimmune diseases have been proposed. HLA class II molecules can trigger autoimmune reactions by altering the functions of T helper (Th) or T regulatory (Treg) lymphocytes. Additionally, *HLA-DR/DQ* alleles can modify antigen recognition in the periphery, which can induce self-directed immune responses [69,70,71,72]. *HLA* class I molecules are involved in the presentation of endogenous antigens, including antigens of viral or bacterial origin, which are known to be important environmental triggering factors for autoimmunity [73,74]. The role of *HLA* in virus-induced autoimmunity should also be taken into account when analyzing associations between HLA and COVID-19 severity. Since the beginning of the pandemic, SARS-CoV-2 infection has been widely reported to be a risk factor for autoimmune diseases, including GD, idiopathic thrombocytopenic purpura (ITP), myasthenia gravis, SLE, and many others [75]. It was hypothesized that superantigen-dependent (Sag) immune dysfunctions are involved in autoimmune processes during COVID-19 [76]. Superantigens are activators of polyclonal lymphocytes, which are resistant to proteases and thermal denaturation [77,78]. Superantigen pathogenic activity associated with its simultaneous binding to the T-cell receptor and MHC class II (less frequently MHC class I) on the antigen presentation cells (APCs) leads to polyclonal activation of T-cells, including autoreactive clones [79]. Very important observations have emerged from studies of COVID-19-related multisystem inflammatory syndrome in children (MIS-C) and adults (MIS-A). It has been postulated that these severe conditions result from autoinflammation and autoimmunity and that potential mechanisms of such reactions involve MHC class II-related immune activation [80]. Moreover, it was speculated that the hyperinflammatory polyclonal T-lymphocyte proliferation in MIS-C was associated with MHC class I molecules, especially HLA-A*02, B*35, and C*04 [81]. Alleles belonging to these allelic groups were associated with a profound expansion of T-cell receptor β variable gene 11-2 (TRBV11-2), which correlated with MIS-C severity [81]. These novel findings further confirm our observation of the relationship between HLA and COVID-19, as well as provide broader evidence that HLA-dependent COVID-19 severity can be significantly associated with HLA-mediated autoimmune reactions.

We observed the phenomenon that the alleles associated with symptomatic COVID-19 did not overlap with alleles associated with the severe course of the disease. This finding can be explained by the fact that the whole group of symptomatic COVID-19 patients (including severe and non-severe courses) was compared to the healthy controls in the first step in order to evaluate susceptibility to COVID-19 and in the second step, this whole group was divided into two smaller groups (severe and non-severe courses) that were then compared to each other. Therefore, one potential reason for the lack of overlap is that the difference in the distribution of alleles between severe and non-severe groups was so large that no allele found to be significant in the whole group achieved statistical significance in the subgroups. The other potential reason that should be taken into account is the fact that subgroups were smaller than the whole group and that there was a domination of severe COVID-19 patients. This domination of severe COVID-19 can be considered a limitation of the study because it did not represent a regular distribution of disease severity in our population; on the other hand, it may be considered a strength of the study as we paid special attention to individuals at high risk of severe COVID-19 course.

The most important limitation of the study is the small sample size, as our analysis included a group of 105 patients with symptomatic COVID-19, which was further divided into SC and non-SC subgroups. Additionally, our database did not include data on pre-existing conditions, which may influence the disease outcomes. Therefore, the obtained results require further confirmation in larger cohorts, especially with regards to alleles with a small number of carriers, mainly B*42:01 and DRB1*11:02 for the risk of symptomatic COVID-19 and DRB1*08:01 for the decreased risk of severe COVID-19.

## 5. Conclusions

The present study has demonstrated significant associations between HLA alleles and risk of symptomatic course of COVID-19 in Caucasians. The symptomatic course of SARS-CoV-2 infection appears to be associated with the presence of HLA-A*30:01, B*44:02, B*52:01, C*05:01, C*17:01, and DRB1*11:02, while HLA-C*07:04 and DQB1*03:03 seem to play a protective role. Moreover, we have demonstrated that the severe course of COVID-19 can be associated with the presence of HLA-B*08:01, C*04:01, DRB1*03:01, and DQB1*03:01, while HLA-DRB1*08:01 appears to be protective. Our study constitutes further evidence that the severe course of COVID-19 can be associated with HLA-mediated autoimmune reactions triggered by the virus. Moreover, the identification of alleles potentially related to symptomatic SARS-CoV-2 infection as well as to the severe course of COVID-19 provides a novel possibility of developing a precise HLA-focused diagnostic tool for the assessment of the risk of severe COVID-19 course in an individual patient. Identification of high-risk groups can enable the introduction of prevention strategies and individualization of treatment modalities in persons with genetic susceptibility to severe disease courses.

## Figures and Tables

**Table 1:** Summary of the results of previous studies with high-resolution (four-digit) methods on HLA impact on COVID-19 risk and course.

Risk and Clinical Course	Allele	Sizes of Compared Groups	*p*-Value	Country [Ref]
Protection	A*03:01	159 vs. 52 *	0.035	Vietnam [29]
	A*30:01	159 vs. 52 *	0.017	Vietnam [29]
	B*14:02	9373 vs. 5943 *	0.006	Spain [22]
	B*51:01	575 vs. 28,927 *	0.0001	Saudi Arabia [24]
	B*51:01	299 vs. 2781 *	<0.036	Armenia [25]
	B*51:01	109 vs. 70 *	0.027	Iran [21]
	C*08:02	9373 vs. 5943 *	0.024	Spain [22]
	E*01:01 + E*01:01	122 vs. 68 *	0.044	Iran [30]
	DPB1*03:01	332 ^+^	0.03	China [31]
	DRB1*11:05	109 vs. 70 *	0.003	Iran [21]
	DRB1*12:01	332 ^+^	0.04	China [31]
	DRB1*13:05	109 vs. 70 *	0.022	Iran [21]
	DRB1*14:01	109 vs. 70 *	0.006	Iran [21]
	DRB1*15:01	159 vs. 52 *	0.009	Vietnam [29]
	DRB5*02:02	159 vs. 52 *	0.004	Vietnam [29]
	DQA1*01:02	159 vs. 52 *	0.031	Vietnam [29]
Asymptomatic	B*15:01	1428 vs. 29,947 *	0.00006	USA [32]
infection	DRB1*04:01	49 vs. 69 ^a^	0.003	UK [33]
Infection	A*30:02	234 vs. 22,000 *	0.0017	USA [34]
(all clinical courses)	B*07:35	109 vs. 70 *	0.031	Iran [21]
	B*27:07	99 vs. 1017 *	0.00001	Italy [35]
	C*04:01	182 vs. 619 *	0.012	Italy (Sardinia) [36]
	DRB1*07:01	109 vs. 70 *	0.003	Iran [21]
	DRB1*08:02	234 vs. 22,000 *	0.01	USA [34]
	DRB1*09:01	159 vs. 52 *	0.044	Vietnam [29]
	DRB1*15:01	99 vs. 1017 *	0.0015	Italy [35]
	DRB1*15:01	575 vs. 28,927 *	<0.0001	Saudi Arabia [24]
	DQB1*06:02	99 vs. 1017 *	0.0001	Italy [35]
Mild disease	A*02:01:01	575 vs. 28,927 *	0.0114	Saudi Arabia [24]
	DRB1*05:01	159 vs. 52 *	0.034	Vietnam [29]
	DPA1*02:01	159 vs. 52 *	0.012	Vietnam [29]
	DRB1*09:01	159 vs. 52 *	0.026	Vietnam [29]
Moderate disease	DPA1*01:03	159 vs. 52 *	0.003	Vietnam [29]
	DRB1*04:01	109 vs. 70 *	0.0002	Iran [21]
Severe disease	A*11:01	9373 vs. 5943 *	0.033	Spain [22]
	A*11:01	137 vs. 53 ^s^	0.015	Japan [37]
	A*11:01	Epi	0.003	Japan [38]
	A*11:01	332 ^+^	0.008	China [31]
	B*50:01	575 vs. 28,927 *	<0.0001	Saudi Arabia [24]
	B*51:01	332 ^+^	0.007	China [31]
	C*04:01	9373 vs. 5943 *	0.045	Spain [22]
	C*04:01	299 vs. 2781 *	<0.021	Armenia [25]
	C*04:01	435 ^+^	0.00011	Germany, Spain, Switzerland, US [23]
	C*04:01	54 vs. 42 ^a^	0.02	India [38]
	C*06:02	575 vs. 28,927 *	<0.0001	Saudi Arabia [24]
	C*14:02	332 ^+^	0.003	China [31]
	E*01:03 + E01:03	122 vs. 68 *	0.02	Iran [30]
	F*01:01	159 vs. 52 *	<0.001	Vietnam [29]
	F*01:03	159 vs. 52 *	0.0028	Vietnam [29]
	DPA1*01:03	54 vs. 42 ^a^	0.001	India [39]
	DPB1*04:01	159 vs. 52 *	0.001	Vietnam [29]
	DRB1*01:01	332 ^+^	0.02	China [31]
	DRB1*04:03	109 vs. 70 *	0.004	Iran [21]
	DRB1*07:01	575 vs. 28,927 *	0.0047	Saudi Arabia [24]
	DRB1*08:01	39 vs. 143	0.024	Italy (Sardinia) [36]
	DRB1*09:01	73 ^s^ vs. 105	0.003	Japan [40]
	DRB1*11:01	109 vs. 70 *	<0.001	Iran [21]
	DRB1*14:04	332 ^+^	0.01	China [31]
	DRB3*01:01	8 ^s^ vs. 8	0.0064	China [41]
	DRB5*01:01	54 vs. 42 ^a^	0.03	India [38]
	DQA1*01:01	332 ^+^	0.04	China [31]
	DQA1*01:02	159 vs. 52 *	0.004	Vietnam [29]
	DQA1*01:03	209 ^+^	0.0013	Japan [42]
	DQA1*03:01	54 vs. 42 ^a^	0.03	India [38]
	DQB1*05:02	159 vs. 52 *	0.008	Vietnam [29]
	DQB1*06:01	209 ^+^	0.013	Japan [41]

Abbreviations: ^a^, asymptomatic COVID-19; Epi, epidemiologic study; ^s^, severe COVID-19; *, controls; ^+^, COVID-19-positive.

**Table 2:** Alleles associated with risk of symptomatic SARS-CoV-2 infection (COVID-19) compared to the control group, presented with odds ratios (ORs).

Allele	Association	Symptomatic COVID-19; *n* = 105 No. of Carriers (%)	Healthy Control; *n* = 2217 No. of Carriers (%)	*p*-Value	Odds Ratio (OR)
A*30:01	risk	8 (7.62%)	66 (2.84%)	0.008	2.7
B*44:02	risk	18 (17.14%)	214 (9.22%)	0.01	1.9
B*42:01	risk	4 (3.81%)	2 (0.09%)	<0.001	43.9
C*05:01	risk	19 (18.1%)	189 (8.14%)	<0.001	2.4
C*17:01	risk	5 (4.76%)	22 (0.95%)	<0.001	5.0
C*07:04	protective	0 (0.0%)	99 (4.26%)	0.02	-
DRB1*11:02	risk	3 (2.86%)	8 (0.34%)	<0.001	8.1
DQB1*03:03	protective	3 (2.86%)	210 (9.04%)	<0.001	0.3

**Table 3:** Alleles associated with risk of severe SARS-CoV-2 infection (COVID-19) compared to non-severe infection, presented with odds ratios.

Allele	Association	Severe COVID-19; *n* = 67 No. of Carriers (%)	Non-Severe COVID-19; *n* = 38 No. of Carriers (%)	*p*-Value	Odds Ratio (OR)
B*08:01	risk	12 (11.43%)	1 (0.04%)	0.02	8.1
C*04:01	risk	16 (15.24%)	3 (0.13%)	0.04	3.7
DRB1*03:01	risk	20 (19.05%)	4 (0.17%)	0.02	3.6
DQB1*03:01	risk	30 (28.57%)	9 (0.39%)	0.03	2.6
DQB1*04:02	risk	7 (6.67%)	0 (0.0%)	0.04	-
DRB1*08:01	protective	1 (0.22%)	5 (0.95%)	0.01	0.1

## Data Availability

The data presented in this study are available on request from the corresponding author due to privacy and ethical restrictions.
